# A Novel Nano-Laminated GdB_2_C_2_ with Excellent Electromagnetic Wave Absorption Performance and Ultra-High-Temperature Thermostability

**DOI:** 10.3390/nano14121025

**Published:** 2024-06-13

**Authors:** Longfei Jiang, Gang Qin, Pengxing Cui, Guoqing Wang, Xiaobing Zhou

**Affiliations:** 1 School of Materials Science and Chemical Engineering, Ningbo University, Ningbo 315211, China; jianglongfei@nimte.ac.cn; 2 Zhejiang Key Laboratory of Data-Driven High-Safety Energy Materials and Applications, Ningbo Key Laboratory of Special Energy Materials and Chemistry, Ningbo Institute of Materials Technology and Engineering, Chinese Academy of Sciences, Ningbo 315201, China; qingang@nimte.ac.cn (G.Q.); cuipengxing@nimte.ac.cn (P.C.); wanggq@nimte.ac.cn (G.W.)

**Keywords:** GdB_2_C_2_, ternary layered materials, electromagnetic wave absorption, ultra-high-temperature ceramic

## Abstract

A novel nano-laminated GdB_2_C_2_ material was successfully synthesized using GdH_2_, B_4_C, and C *via* an in situ solid-state reaction approach for the first time. The formation process of GdB_2_C_2_ was revealed based on the microstructure and phase evolution investigation. Purity of 96.4 wt.% GdB_2_C_2_ was obtained at a low temperature of 1500 °C, while a nearly fully pure GdB_2_C_2_ could be obtained at a temperature over 1700 °C. The as-obtained GdB_2_C_2_ presented excellent thermal stability at a high temperature of 2100 °C in Ar atmosphere due to the stable framework formed by the high-covalence four-member and eight-member B-C rings in GdB_2_C_2_. The GdB_2_C_2_ material synthesized at 1500 °C demonstrated a remarkably low minimum reflection loss (RL_min_) of −47.01 dB (3.44 mm) and a broad effective absorption bandwidth (EAB) of 1.76 GHz. The possible electromagnetic wave absorption (EMWA) mechanism could be ascribed to the nano-laminated structure and appropriate electrical conductivity, which facilitated good impedance matching, remarkable conduction loss, and interfacial polarization, along with the reflection and scattering of electromagnetic waves at multiple interfaces. The GdB_2_C_2_, with excellent EMWA performance as well as remarkable ultra-high-temperature thermal stability, could be a promising candidate for the application of EMWA materials in extreme ultra-high temperatures.

## 1. Introduction

Ultra-high-temperature ceramics (UHTCs) are materials which are usually used at temperatures above 1800 °C. Most UHTCs, including transition-metal borides and/or carbides, are characterized by their strong covalent bonds [1,2,3]. Due to the robust covalent bonds between the transition metal and the boron and/or carbon, the family of UHTCs boasts an array of unparalleled characteristics, including elevated melting points, superior hardness, outstanding mechanical performance at high temperatures, remarkable thermal stability, and commendable resistance to both oxidation and corrosion [4,5,6,7]. Therefore, UHTCs are promising candidates for aerospace applications, including nose cone caps and leading edges, and coatings for the protection of high-temperature structural components in hypersonic vehicles, e.g., coatings of carbon fiber-reinforced ceramic matrix composites which improve the synergistic blend of strength and durability [8,9].

On the other hand, for aerospace applications, UHTCs not only should have high-temperature thermal stability but should also possess desirable functional capabilities, such as excellent electromagnetic wave absorption (EMWA) performance [10,11,12]. Numerous EMWA materials have been investigated, such as carbon-based materials [13,14,15,16], magnetic metal materials [17,18], ferrite, as well as its composites [19], and polymer matrix composites [20]. Magnetic materials such as ferrite exhibit excellent EMWA performance, but they lack dielectric loss capacity, and once they exceed the Curie temperature, their magnetic properties disappear, rendering the magnetic loss mechanism ineffective [19]. Polymer-based composites are lightweight and have high design flexibility. However, the materials in question possess relatively low melting points, rendering them ill-suited for high-temperature applications [20,21]. In addition, there are carbon-related materials such as carbon fiber, carbon nanotubes, and graphene that exhibit the characteristics of reduced weight, lower density, enhanced electrical conductivity, and superior mechanical properties [14,15,16,22,23,24,25,26,27,28,29]. Nevertheless, these materials often present a high dielectric constant coupled with low permeability, which compromises impedance matching and restricts the penetration of electromagnetic waves into the material. Additionally, their susceptibility to oxidation in high-temperature environments renders them unsuitable for applications at elevated temperatures [30]. The task of creating a material with a relatively broad effective absorption bandwidth and enhanced EMWA performance poses a formidable challenge. Additionally, these materials must exhibit low density, thin thickness, and exceptional thermal stability even under extreme temperatures, such as stealth materials used in high-speed military vehicles [31].

Rare earth diborocarbides (REB_2_C_2_, RE = Sc, Y, and lanthanide elements) are a group of laminated structure materials like MAX phases (M represents a transition metal; A denotes elements from groups IIIA, IVA, VA, or VIA; and X stands for carbon or nitrogen) [32,33,34,35]. Within the rare earth diborocarbides, GdB_2_C_2_ belongs to a tetragonal structure with the *P*4/*mbm* space group (No.127) [36]. Gd atoms are configured in alternating B-C layers along the z-axis, Gd-Gd bonds are metallic bonds, while B-C bonds are covalent bonds, leading to the formation of four-membered and eight-membered B-C rings. The anisotropy structures of chemical bonds may result in GdB_2_C_2_ showing strong anisotropy in physical properties [37,38]. For example, with regard to the magnetic properties of GdB_2_C_2_, it was demonstrated that it was antiferromagnetic in the *c* plane, while it was ferromagnetic along the *c* axis [39]. The resistivities of GdB_2_C_2_ were reported to decrease with decreasing temperature, which showed metallic conductivity and electron-type conductors [40].

On the other hand, most of the reported synthesis methods for REB_2_C_2_ have been related to YB_2_C_2_ [41,42]. There are few studies on fabrication methods for GdB_2_C_2_ [40]. Single-crystal GdB_2_C_2_ was synthesized employing Gd, B, and C as starting materials using repetitive arc-melting. The samples were then encapsulated in a vacuum-sealed silica tube for a duration of several days [36]. The other representative synthesis procedure for GdB_2_C_2_ was a two-step procedure, including GdB_4_ fabricated by induction heating mixtures of Gd and B at 1900 °C. Then, GdB_4_ and graphite were heated at 1900 °C for three hours to obtain GdB_2_C_2_ [40]. Additionally, to the best of the authors’ knowledge, there are limited reports on the one-step synthesis of GdB_2_C_2_ powders, their EMWA properties, and their thermal stability at ultra-high temperatures. Given the typical nano-laminated structure of GdB_2_C_2_, which is akin to MAX phases, it is anticipated to exhibit exceptional EMWA performance due to the potential multiple interface scattering losses and dielectric loss mechanisms inherent in GdB_2_C_2_ [32,33,43,44,45,46].

Therefore, the main aim of this work was to develop a facile one-step fabrication method for the nano-laminated GdB_2_C_2_ material and reveal the EMWA mechanism. In addition, the thermal stability at an ultra-high temperature of 2100 °C was investigated to determine the potential aerospace applications of GdB_2_C_2_.

## 2. Experimental Procedure

### 2.1. Materials

GdH_2_, B_4_C, and carbon black were employed as raw materials for synthesizing GdB_2_C_2_. The GdH_2_ powders (purity > 99.9%, average particle size: ~70 μm) were commercially supplied by Hunan Rare Earth Metal Materials Research Institute Co., Ltd., Changsha, China. The B_4_C powders, with a purity of 99% and a mean particle size of around 500 nanometers, were procured from the Suzhou Nutpool Materials Technology Co., Ltd., Jian, China. Carbon black powders, characterized by a purity of 99.9% and a mean particle size of 500 nanometers, were acquired from ENO High-Tech Material Co., Ltd., Qinhuangdao, China.

### 2.2. Fabrication of GdB_2_C_2_

**Figure 1:**
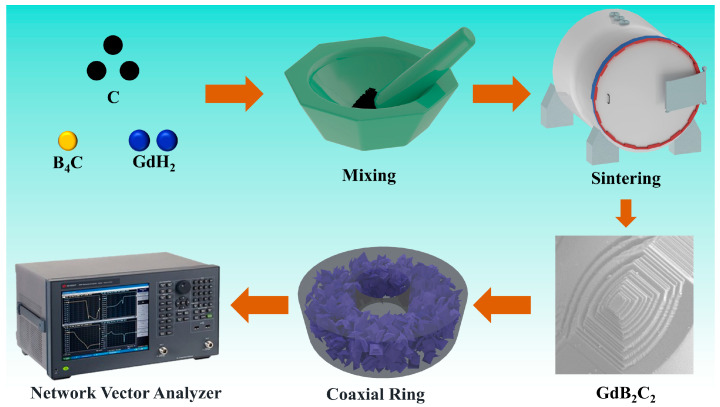
Schematic of GdB_2_C_2_ powder synthesis procedures and EMWA property test.

The GdH_2_, B_4_C and carbon black powders were mixed in a glovebox under an argon atmosphere with a molar ratio of GdH_2_:B_4_C:C = 2:1:3. To investigate the in situ reaction process of GdB_2_C_2_, the mixed powders were fired at various temperatures ranging from 900 °C to 1800 °C for 4 h in a graphite furnace under an argon atmosphere. The heating and cooling rates were 5 °C/min. The as-obtained powders were ground in an agate mortar for 40 min. Figure 1 shows the schematics of the GdB_2_C_2_ powder synthesis procedures.

### 2.3. Characterizations

The compositions of phases and crystalline structures within the samples synthesized at different temperatures were evaluated using a Bruker AXS D8 Advance X-ray diffractometer, procured from Rheinstetten, Germany, which operates on Cu Kα radiation with a wavelength set at λ = 1.5406 Å. The power parameters for this instrument were set at 1600 W, equating to a current of 40 mA and a voltage of 40 kV, while utilizing a step scan methodology of 0.02°/2θ with a step duration of 0.2 s. The constituents of the phases and parameters of the lattice within the resulting materials were deciphered through the Rietveld refinement processing of the XRD patterns, facilitated by TOPAS-Academic v6 software. The microstructures of the samples synthesized at different temperatures were examined using a scanning electron microscope (SEM; Regulus 8230, Hitachi, Tokyo, Japan) equipped with an energy-dispersive spectroscopy (EDS) system. The average grain sizes were quantified by analyzing a selection of SEM micrographs, with a rigorous count of no less than 100 grains per sample. Transmission electron microscopy (TEM; Talos F200x, Thermo Fisher Scientific, Waltham, Massachusetts, USA) was employed to confirm the microstructure and phase composition of the GdB_2_C_2_ synthesized at 1500 °C. Thin foil of the sample for TEM analysis was fabricated using the focused ion beam (FIB) technique (Aurgia, Carl Zeiss, Thornwood, NY, USA).

To investigate the effects of the heat-treated temperatures on the EMWA performance, the complex permittivity and complex permeability of the samples fabricated at 1500 and 1800 °C were measured at a frequency range from 2 to 18 GHz using a Keysight E5063A Network Analyzer (Santa Rosa, California, USA). A toroidal ring was fabricated using the as-obtained GdB_2_C_2_ powders mixed with 60 vol.% paraffin for measurement of the complex permittivity and complex permeability. The geometry size of the toroidal ring was 3 mm (inner diameter), 7 mm (outer diameter), and 2 mm (thickness). A start frequency of 2 GHz, an intermediate frequency bandwidth of 300 Hz, and 201 sample points were set during measurement. Following port and path calibrations, system accuracy was verified using air and polytetrafluoroethylene standards. At least five samples were measured for each synthesized temperature, and the mean value was calculated.

## 3. Results and Discussion

### 3.1. Microstructure and Phase Composition of GdB_2_C_2_

**Figure 2:**
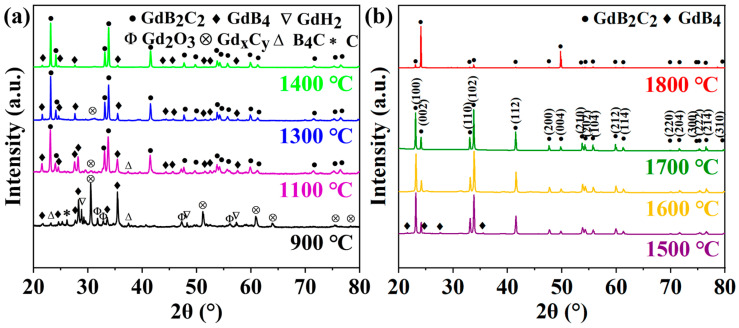
XRD patterns of GdB_2_C_2_ fabricated at different temperatures: (**a**) 900–1400 °C, (**b**) 1500–1800 °C.

Figure 2 presents the X-ray diffraction (XRD) patterns of the powders synthesized at various temperatures ranging from 900 to 1800 °C. The GdB_4_, Gd-C, and Gd_2_O_3_ phases were detected at the temperature of 900 °C, in addition to the residual un-reacted raw materials of GdH_2_, B_4_C, and C. As the synthesis temperature increased to 1100–1300 °C, GdB_2_C_2_ was formed, while the GdB_4_ and Gd-C phases were still detected, which implied that the reaction was not completed. When the temperature increased to 1400–1500 °C, GdB_2_C_2_ was the main phase, and just a small amount of the GdB_4_ impurity phase was detected. While the temperature increased to 1600–1800 °C, nearly fully pure GdB_2_C_2_ was obtained. 

**Figure 3:**
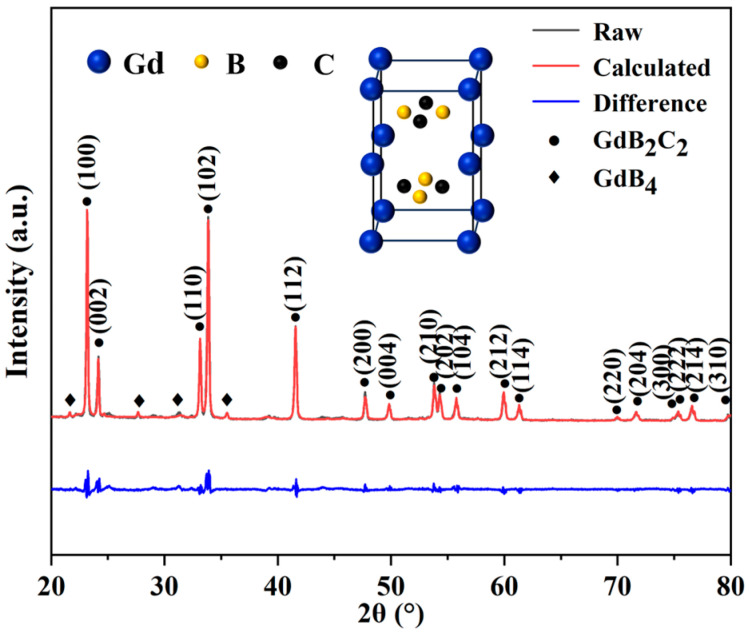
Rietveld refinement of XRD pattern of GdB_2_C_2_ synthesized at 1500 °C.

**Figure 4:**
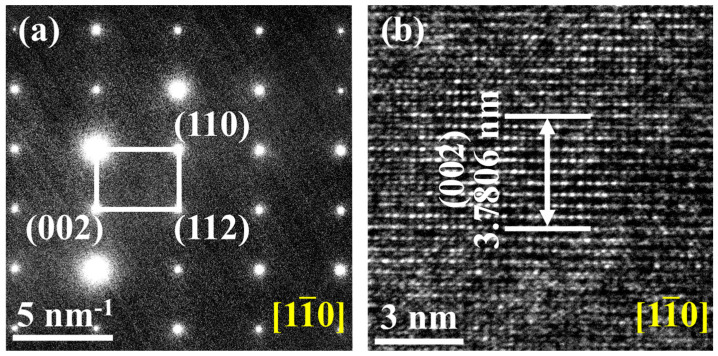
(**a**) SAED pattern and (**b**) HR-TEM image of GdB_2_C_2_ synthesized at 1500 °C.

According to the Rietveld refinement, the content of GdB_2_C_2_ phase was increased from 95.47 wt. % to 100 wt % as the synthesis temperature increased from 1400 to 1700 °C, while the content of GdB_4_ impurity decreased from 4.53 wt% to 0 wt%. Figure 3 shows the typical Rietveld refinement of the XRD pattern of GdB_2_C_2_ fabricated at 1500 °C. The Rietveld fitting results (reliability factors R_wp_ = 9.4%) indicated that a tetragonal structure of GdB_2_C_2_ was obtained (the inset in Figure 3). An R_wp_ value is a critical metric for evaluating the fit between calculated and experimental diffraction patterns. An R_wp_ value that is lower than 10% indicates the reliability of the Rietveld fitting results. The lattice parameters *a* and *c* were determined to be approximately 3.78 Å and 7.27 Å (Table 1), which were almost equal to the reported lattice parameters of GdB_2_C_2_ [40]. The lattice parameter measurements were validated by the TEM analysis, which confirmed the *c*-axis value of 7.5612 Å in the GdB_2_C_2_ powders through selected area electron diffraction (SAED) and high-resolution transmission electron microscopy (HR-TEM) imaging. These observations are presented in Figure 4a,b. The HR-TEM image elucidates the atomic arrangement along the zone axis, revealing a lattice fringe spacing of 0.37806 nm, which corresponds to the (002) plane of GdB_2_C_2._ The experimentally determined spacing of the (002) plane (Figure 4b) thereby substantiated the successful synthesis of the GdB_2_C_2_. It is noted that there was some difference in the value of the lattice constant *c* determined from X-ray measurements (0.727 nm) and TEM analysis (0.75612 nm). This may due to the TEM image only providing localized measurements, which may be influenced by the localized stresses and/or lattice distortions.

**Figure 5:**
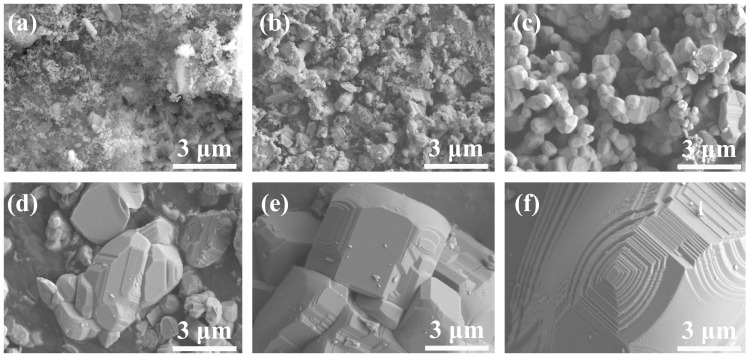
SEM images of GdB_2_C_2_ powders fabricated at varying temperatures: (**a**) 1300 °C, (**b**) 1400 °C, (**c**) 1500 °C, (**d**) 1600 °C, (**e**) 1700 °C, and (**f**) 1800 °C.

**Figure 6:**
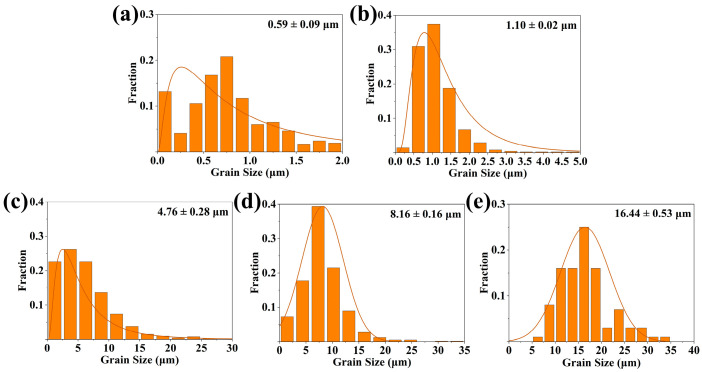
Particle size distribution of GdB_2_C_2_ powders fabricated at varying temperatures obtained by SEM image analysis: (**a**) 1400 °C, (**b**) 1500 °C, (**c**) 1600 °C, (**d**) 1700 °C and (**e**) 1800 °C.

According to the phase evolution analysis of the samples fabricated at various temperatures, the formation process of GdB_2_C_2_ via the in situ reaction among GdH_2_, B_4_C, and C could be concluded as follows: At the low temperature of 900 °C, GdH_2_ underwent dehydrogenation and decomposed to Gd and H_2_ (reaction 1) [47]. The generated Gd diffused on the surfaces of B_4_C and C and reacted with them to form GdB_4_ and Gd-C phases (reaction 2). At the temperature ranging from 1100 to 1300 °C, the intermediate phases of GdB_4_ and Gd-C further reacted with residual C, and GdB_2_C_2_ began to be formed (reaction 3). The grain size was only several hundred nanometers due to the low synthesis temperature (Figure 5a). Upon elevating the temperature to 1400 °C, most of the GdB_4_ and Gd-C intermediate phases underwent transformation into GdB_2_C_2_. The predominant size of the GdB_2_C_2_ particles ranged from 0.5 to 1 μm, while some nano-sized particles were also discerned (Figure 5b and Figure 6a). When the temperature increased to 1500 °C, the GdB_2_C_2_ grains growth along with the nano-sized particles disappeared (Figure 5c). The mean particle size was around 1.1 μm (Figure 6b). As the temperature increased to over 1600 °C, almost pure GdB_2_C_2_ was obtained. At this high temperature range, the GdB_2_C_2_ grains grew rapidly. The mean particle size increased from 4.76 μm to 16.44 μm as the temperature increased from 1600 to 1800 °C (Figure 6c–e). The typical nano-laminated structure of GdB_2_C_2_ was observed (Figure 5d–f). It is noted that the GdB_2_C_2_ grains preferred to grow along the *c* plane; as a result, the (002) diffraction peak was emphasized as the highest peak for the sample fabricated at 1800 °C (Figure 2b). This phenomenon was also observed in the single-crystal GdB_2_C_2_ [38].

GdH_2_ → Gd + H_2_ (1)

2Gd + B_4_C + C → GdB_4_ + GdC_2_ (2)

GdC_2_ + GdB_4_ + 2C → 2GdB_2_C_2_ (3)

### 3.2. Ultra-High-Temperature Thermal Stability of GdB_2_C_2_ at 2100 °C

**Figure 7:**
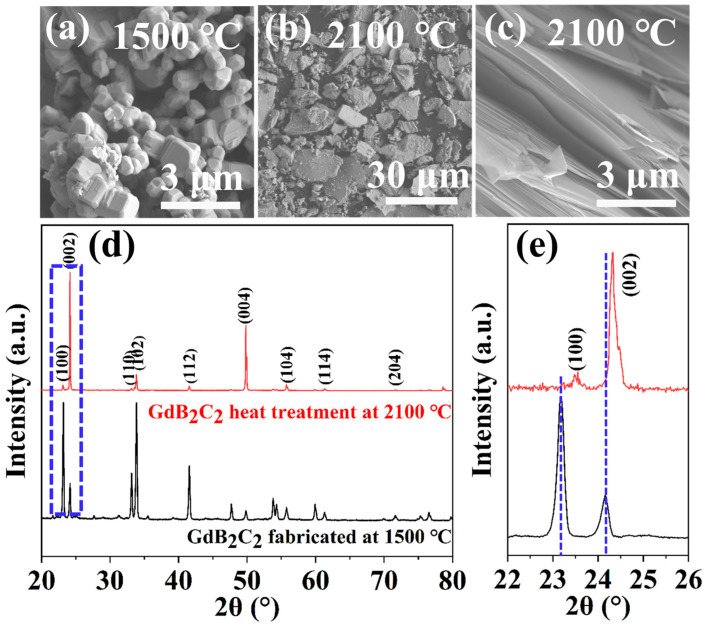
SEM images of GdB_2_C_2_ powders at (**a**) 1500 °C and heat treatment at (**b**,**c**) 2100 °C, (**d**) XRD pattern of the as-synthesized GdB_2_C_2_ at 1500 °C and the sample after heat treatment at 2100 °C, (**e**) partial XRD patterns showing a peak shift of (002).

Thermal stability at high temperature is a critical property in the application of UHTCs. Figure 7a–c show the morphology of GdB_2_C_2_ powders (prepared at 1500 °C) after heat treatment at 2100 °C for 20 min under argon atmosphere. Obvious grain growth was observed. The maximum length of GdB_2_C_2_ was around 30 μm, which was significantly increased and was up to 30 times larger than that of the original as-synthesized GdB_2_C_2_ sample (~1.1 μm). The SEM image of the fracture surface (Figure 7c) reveals a characteristic nano-laminated structure akin to that of MAX phases. The observed laminated fracture features, including delamination, slipping, and kink band formation (Figure 7c), indicate the ductile behavior of GdB_2_C_2_.

The phase composition of GdB_2_C_2_ after being heat-treated at 2100 °C is depicted in Figure 7d,e. No significant phase transition was observed. However, GdB_2_C_2_ post- heat-treated at 2100 °C exhibits strong diffraction peaks corresponding to the (002) and (004) crystallographic planes. Additionally, the characteristic diffraction peaks of the (100) and (002) planes for the heat-treated sample at 2100 °C exhibit a shift towards higher 2 theta angles when contrasted with the as-obtained GdB_2_C_2_ powders. This implied that the lattice parameters of dB_2_C_2_ after heat treatment at 2100 °C decreased compared to the as-obtained GdB_2_C_2_ powders.

The decrement in the lattice parameters after heat treatment can be attributed to the volatilization of Gd atoms, which resulted in the formation of Gd vacancies. This can be easily explained by the crystal structure of GdB_2_C_2_, which had Gd-B and Gd-C bonds with bond lengths of 276.4 and 272.0 pm, respectively, as well as shorter B-C bonds (162.2 pm and/or 151.2 pm) [36]. The bond energies of the Gd-B and Gd-C interactions were notably lower than those of the B-C bonds, rendering the former more susceptible to dissociation at the elevated temperature of 2100 °C. Consequently, Gd atoms preferentially evaporated from the surface of the material. The evaporation of Gd atoms and the consequent vacancies naturally resulted in a contraction of the lattice as the remaining atoms repositioned to maintain the integrity of the crystal structure. On the other hand, the robustly covalent four-membered and eight-membered B-C rings were preserved, resisting evaporation. This suggested that the high-covalence four-member and eight-member B-C rings play a critical role in the ultra-high-temperature thermal stability of GdB_2_C_2_. Comparable behavior has been noted in analogous YB_2_C_2_ materials, further supporting the significance of these high-covalence B-C rings in maintaining the thermal stability of the related compounds [42].

### 3.3. Electromagnetic Wave Absorption Properties of GdB_2_C_2_

The interaction of a material with EMW is determined by various factors, with the efficiency of impedance matching playing a crucial role. Effective impedance matching enhances the penetration and absorption of the EMW into the material, whereas inadequate matching results in EMW reflection. The complex dielectric constant and complex permeability reflect the aptitude of a material for absorbing EMW, which in turn determines its efficiency in transforming them into alternative forms of energy [48]. 

To investigate the effects of fabrication temperature on the EMWA performance of the GdB_2_C_2_ powders, the complex permittivity and complex permeability of the GdB_2_C_2_ powders synthesized at 1500 and 1800 °C were measured. It was observed that the real and imaginary parts of the complex permeability remained nearly constant at approximately 1 and 0 at the frequency range of 2–18 GHz, respectively. This indicates that the magnetic loss in these materials was negligible. Consequently, the EMWA properties of the GdB_2_C_2_ powders were predominantly governed by complex permittivity [49,50].

**Figure 8:**
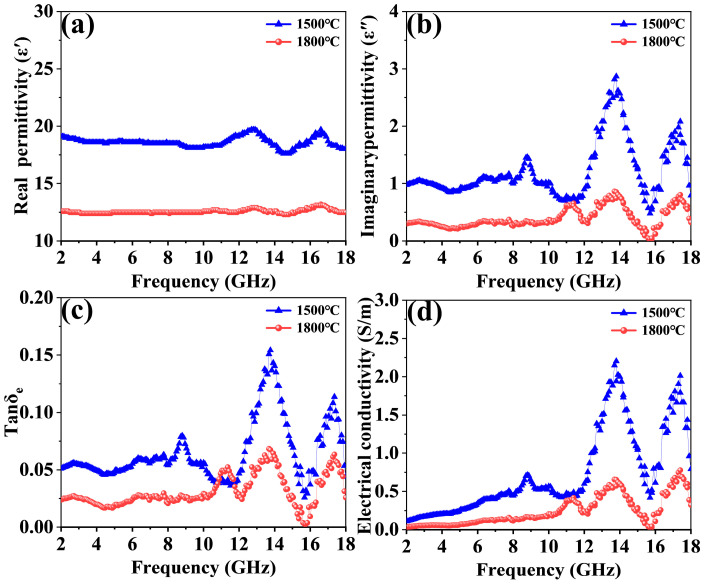
Real (**a**) and imaginary (**b**) parts of the complex permittivity, dielectric loss angle (**c**), and electrical conductivity (**d**) of GdB_2_C_2_ synthesized at 1500 °C and 1800 °C.

Figure 8 presents the real (ε′) and imaginary (ε″) parts of the complex permittivity of the GdB_2_C_2_ powders synthesized at 1500 and 1800 °C. Conventionally, ε′ is indicative of the storage capability of dielectric energy, whereas ε″ reflects the dissipation of dielectric energy [51,52]. Furthermore, tan δ_e_ (tan δ_e_ = ε′/ε″) is usually used to analyze the dielectric attenuation of a sample, which reflects the EMW attenuation capability of EMWA materials (Figure 8c). The dielectric loss tangent provides a quantitative measure of the efficiency with which the material can attenuate incident EMWs.

Both the *ε*′ and *ε*″ of the GdB_2_C_2_ powders synthesized at 1500 °C were higher than those of the samples synthesized at 1800 °C, confirming that the fine particle size could promote the dielectric properties of GdB_2_C_2_. The finer the grain size, the more interfaces were formed between GdB_2_C_2_ powders and the paraffin matrix. The improvement in the interaction between the GdB_2_C_2_–paraffin and the enhancement of interfacial polarization contributed to the higher *ε*′ of GdB_2_C_2_ powders synthesized at 1500 °C compared to the sample synthesized at 1800 °C. The increase in *ε*″ was primarily attributed to the rise in electrical conductivity. The electrical conductivity can be determined using Equation (4) as follows [53]:

(4) $$\sigma =2\pi \varepsilon_{0}\varepsilon^{\prime \prime }$$

As presented in Figure 8d, the electrical conductivities of GdB_2_C_2_ powders synthesized at 1500 °C were higher than that of the sample synthesized at 1800 °C. This can be mainly attributed to the metallic conductivity characteristic of the laminated structural GdB_2_C_2_ powders, as the finer grain size more easily formed a conductive net structure, which increased the transmission channels of carriers. The diminutive grain size paved the way for the emergence of a conductive network, empowering more expeditious pathways for the conveyance of charge carriers [54]. Furthermore, the *ε*′ and *ε*″ of GdB_2_C_2_ presented a fluctuation corresponding to the resonance. The permittivity of the GdB_2_C_2_ showed typical nonlinear resonant characteristics, indicating the existence of polarization and relaxation behavior, which implied good dielectric loss performance in the corresponding frequency range. In addition, the enhanced number of heterogeneous interfaces caused by the smaller grain size also improved the relaxation process of GdB_2_C_2_, which generated the improvement in relaxation polarization.

Reflection loss (RL) and EAB were used to evaluate the EMWA properties of the as-obtained GdB_2_C_2_ powders synthesized at 1500 and 1800 °C. According to the classic transmission line theory, the RL values of the as-obtained GdB_2_C_2_ powders could be calculated by Equations (7)–(9) [55,56,57] as follows:

(5) $$RL\left(\mathrm{dB}\right)=20\log\left|\left(Z_{\mathrm{in}}-Z_{0}\right)/\left(Z_{\mathrm{in}}+Z_{0}\right)\right|$$

(6) $$Z_{\mathrm{in}}=Z_{0}\sqrt{\mu_{r}/\varepsilon_{r}}\tan h\left[j\left(2\pi \mathrm{fd}/c\right)\sqrt{\mu_{r}\varepsilon_{r}}\right]$$

(7) $$Z_{0}=\sqrt{\mu_{r}/\varepsilon_{r}}$$

**Figure 9:**
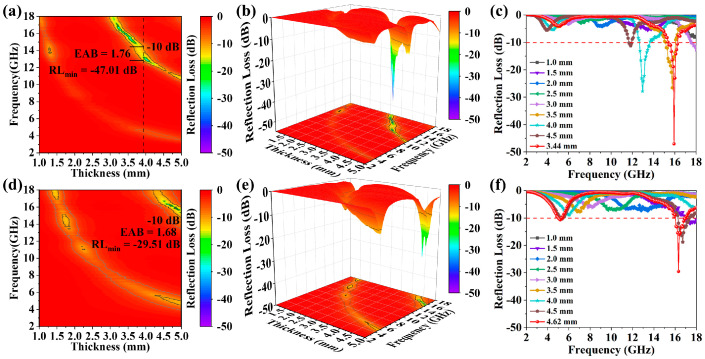
Three-dimensional and two-dimensional patterns of reflection loss values at frequency range of 2 to 18 GHz for different thicknesses of GdB_2_C_2_ samples synthesized at 1500 °C (**a**–**c**) and 1800 °C (**d**–**f**).

Within the equation, Z_0_ stands for the impedance of free space, with Z_in_ embodying the input impedance. The complex relative permeability, μ_r_, unfolds as μ′–jμ″, and the complex relative permittivity, ε_r_, is articulated by ε′–jε″. The symbol c embodies the speed of light, d signifies the thickness of the material, and f epitomizes the frequency. Figure 9 showcases the reflection loss (RL) values for GdB_2_C_2_ powders forged at 1500 °C and 1800 °C across the expansive frequency realm of 2–18 GHz with varying thicknesses. The minimum reflection loss (RL_min_) value of the GdB_2_C_2_ synthesized at 1500 °C was −47.01 dB at the frequency of 15.92 GHz with a thickness of 3.44 mm. Meanwhile, for the sample synthesized at 1800 °C, the RL_min_ value was −29.51 dB at the frequency of 16.32 GHz with a large thickness of 4.62 mm. For the sake of comparison, the selected theoretical calculated RL of the as-obtained GdB_2_C_2_ powders synthesized at 1500 and 1800 °C in the frequency range of 2–18 GHz with different thicknesses is shown in Figure 9c,f. The EAB of GdB_2_C_2_ powders synthesized at 1500 °C was wider than that of GdB_2_C_2_ powders synthesized at 1800 °C. The widest EAB was around 1.76 GHz for a thin GdB_2_C_2_ sample with the thickness of 3.86 mm (Figure 9c). Meanwhile, the widest EAB of GdB_2_C_2_ powders synthesized at 1800 °C was merely 1.68 GHz for the sample even with a thickness of 4.96 mm (Figure 9f). This indicates that GdB_2_C_2_ powders synthesized at a low temperature of 1500 °C with a finer grain size can significantly improve the EMWA properties of GdB_2_C_2_.

**Figure 10:**
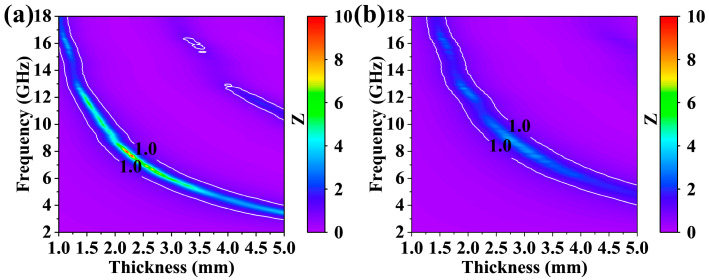
Two-dimensional patterns of Z values of GdB_2_C_2_ samples synthesized at (**a**) 1500 °C and (**b**) 1800 °C.

In order to reveal the intrinsic reason for the improved EMWA performance for the GdB_2_C_2_ powders synthesized at 1500 °C, the impedance match (IM, Z) and attenuation ability were calculated. Figure 10 illustrates the IM (Z) values of these two samples, which were calculated using the following formula [58]:

(8) $$Z=\left|Z_{in}/Z_{0}\right|=\sqrt{\mu_{r}/\varepsilon_{r}}\tanh\left[\;j\left(2\pi fd/c\right)\sqrt{\mu_{r}\varepsilon_{r}\;}\;\right]$$

IM is a critical factor in determining the entry of EMWs into a material’s surface. The optimal IM occurs when the input impedance (*Z_in_*) closely matches the air impedance (*Z*_0_). The closer the IM value *Z* is to 1, the more electromagnetic waves can enter the EMW absorber, and the better impedance it has. The frequency range with good impedance matching of the GdB_2_C_2_ powders synthesized at 1500 °C was larger than that of the sample synthesized at 1800 °C. Therefore, the EMWs could enter into the sample synthesized at 1500 °C, while most of the EMWs were reflected in the case of the sample synthesized at 1800 °C due to the poor IM. 

In addition to evaluating the attenuation ability of the EMW energy of the samples, the attenuation constant (α) of the as-obtained GdB_2_C_2_ powders synthesized at 1500 and 1800 °C in the frequency range of 2–18 GHz was calculated by the following formula [59]:

(9) $$\alpha =\frac{\sqrt{2}\pi f}{c}\sqrt{(\mu^{\prime \prime }\varepsilon^{\prime \prime }-\mu^{\prime }\varepsilon^{\prime })+\sqrt{{\left(\mu^{\prime }\varepsilon^{\prime \prime }+\mu^{\prime \prime }\varepsilon^{\prime }\right)}^{2}+{\left(\mu^{\prime \prime }\varepsilon^{\prime \prime }-\mu^{\prime }\varepsilon^{\prime }\right)}^{2}}}$$

**Figure 11:**
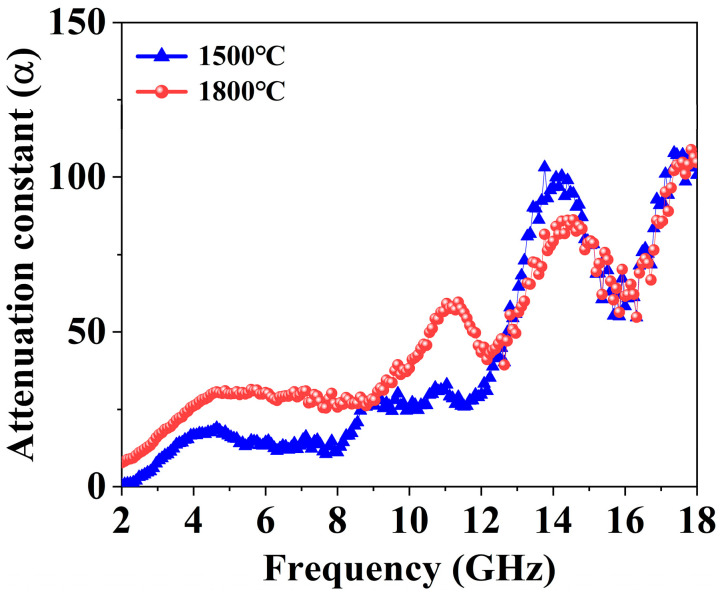
Attenuation constant of GdB_2_C_2_ samples synthesized at 1500 °C and 1800 °C at the frequency range from 2 to 18 GHz.

The elevation of the α value signifies a heightened capacity of the material to effectively attenuate electromagnetic waves [60]. As depicted in Figure 11, samples prepared at 1500 °C exhibited a higher attenuation constant than those prepared at 1800 °C within the frequency range of 12.64 to 18 GHz. Combined with the calculated IM value, the finer grain size of the sample synthesized at 1500 °C improved the IM as well as the attenuation ability compared to the sample synthesized at 1800 °C. As a result, the EMWA performance was augmented. On the other hand, high-performance EMWA materials, such as SiC, ferrite, polymer, and carbon-based materials could be introduced into GdB_2_C_2_ to construct a hybrid multi-phase composite to further improve the EMWA performance of GdB_2_C_2_ and broaden the EAB [61,62,63].

**Figure 12:**
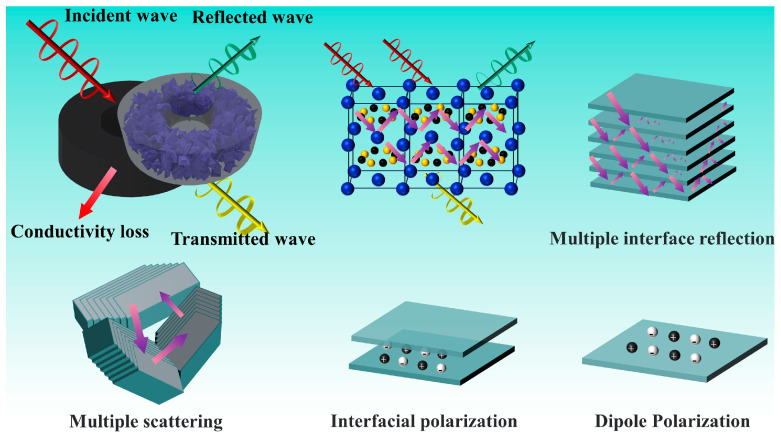
Main EMWA mechanisms of GdB_2_C_2_.

Figure 12 shows the possible EMWA mechanism of the nano-laminated GdB_2_C_2_. Firstly, the optimal IM facilitated the penetration of a substantial portion of EMWs into the GdB_2_C_2_ sample. This is the premise of the excellent EMWA performance of GdB_2_C_2_. Secondly, the metallic conductivity of GdB_2_C_2_ played a pivotal role in inducing conductance loss through electron transition pathways within the material. Thirdly, a large number of homogeneous interfaces and heterogeneous interfaces in the nano-laminated GdB_2_C_2_ sample, such as GdB_2_C_2_/GdB_2_C_2_, GdB_2_C_2_/paraffin, and GdB_2_C_2_/GdB_4_, remarkably increased the interfacial polarization as well as hopping electrons between GdB_2_C_2_ nano-sheets. This was beneficial to improving the dielectric loss of the GdB_2_C_2_. Finally, the two-dimensional nano-laminated GdB_2_C_2_ could form a three-dimensional microstructure and constructed an effective conductive network, resulting in the enhancement of multiple scattering and reflections. Therefore, the excellent EMWA performance of GdB_2_C_2_ was contributed to the synergistic effect of favorable IM, enhanced conductance loss, interfacial polarization, dipole polarization, and multiple scattering and multiple interface reflections.

**Figure 13:**
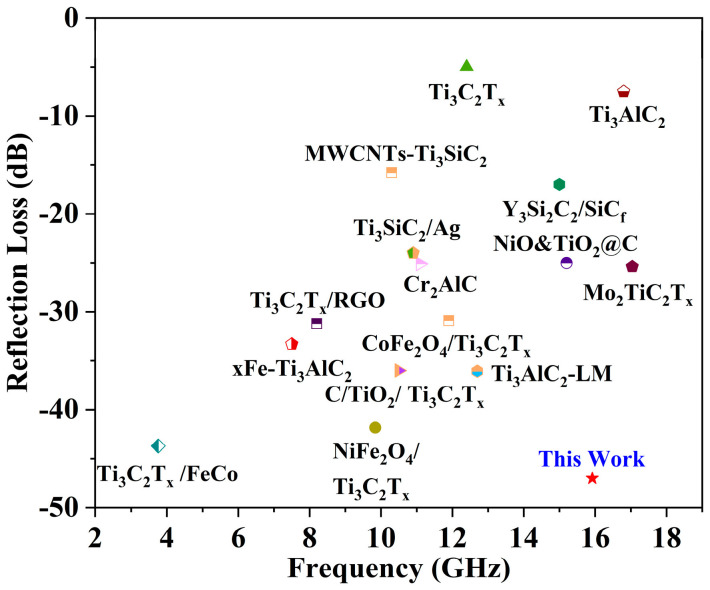
Comparison of EMWA performance of GdB_2_C_2_ with that of other absorbing materials [11,51,54,64,65,66,67,68,69,70,71,72,73,74].

Figure 13 shows the optimal EMWA performance of ternary-layered-structure Ti_3_SiC_2_, Ti_3_AlC_2_, Ti_3_C_2_T_x_, and Ti_3_C_2_T_x_-based materials reported in the relevant literature. Numerous layered-structure materials have been thoroughly investigated, and excellent EMWA properties have been achieved in terms of filler loading, thinner matching thickness, low RL_min_ value, and broader EAB. In this study, GdB_2_C_2_ powders fabricated at 1500 °C presented excellent EMWA properties along with remarkable thermal stability at an ultra-high temperature of 2100 °C. It is noted that all of the EMWA performances of the samples were measured at room temperature. The high-temperature EMWA performance of GdB_2_C_2_ materials will be investigated in future work.

## 4. Conclusions

In conclusion, a novel nano-laminated GdB_2_C_2_ was successfully synthesized using in situ solid-state reaction technology. The formation mechanism of GdB_2_C_2_ was determined based on the investigation of the microstructure and phase composition of the samples synthesized at temperatures ranging from 900 °C to 1800 °C. GdB_2_C_2_ can be initially formed at 1100 °C. GdB_2_C_2_ with purity of 96.4 wt.% was obtained at 1500 °C, while nearly fully pure GdB_2_C_2_ could be fabricated at a temperature over 1700 °C. It is noted that the GdB_2_C_2_ grains preferred to grow along the *c* plane at temperatures over 1800 °C. In addition, the as-obtained GdB_2_C_2_ showed excellent thermal stability at a high temperature of 2100 °C in Ar atmosphere. This can be ascribed to the high-covalence four-member and eight-member B-C rings of GdB_2_C_2_, which formed a stable framework. A comparison of EMWA performance in the GdB_2_C_2_ samples prepared at 1500 °C and 1800 °C was carried out. The minimum reflection loss value (RL_min_) of −47.01 dB with an effective absorption bandwidth (EAB) of 1.76 GHz at a thickness of 3.44 mm was obtained for the GdB_2_C_2_ synthesized at 1500 °C. The main mechanisms responsible for the excellent EMWA performance of GdB_2_C_2_ were good impedance matching, remarkable conduction loss, interfacial polarization, as well as multiple interface reflection and scattering. The excellent EMWA performance as well as the remarkable ultra-high-temperature thermal stability of the as-obtained GdB_2_C_2_ with make it a promising candidate as a next-generation high-temperature EMWA material.

## Figures and Tables

**Table 1:** The R_wp_, lattice parameters (*a* and *c*), and amounts of GdB_2_C_2_ and GdB_4_ phase in the as-obtained powders fabricated at various temperatures according to Rietveld refinement results.

Holding Temperature (°C)	R_wp_	Experimental		GdB_2_C_2_ (wt.%)	GdB_4_ (wt.%)
		*a* (Å)	*c* (Å)		
1400	8.4	3.78	7.28	95.47	4.53
1500	9.4	3.78	7.27	96.38	3.62
1600	8.4	3.79	7.27	99.24	0.76
1700	8.3	3.79	7.27	100	0

## Data Availability

Data are contained within the article.
